# Supplementary material for: Isoflurane Anesthesia’s Impact on Gene Expression Patterns of Rat Brains in an Ischemic Stroke Model
Source: Genes (Basel). 2023 Jul 14;14(7):1448. doi: 10.3390/genes14071448 (PMC10379230; doi:10.3390/genes14071448)
Supplement: Supplementary file 1 [file genes-14-01448-s001.zip › Supplementary Figure S1.pdf]

Figure S1. Experimental design of tMCAO model in rats.

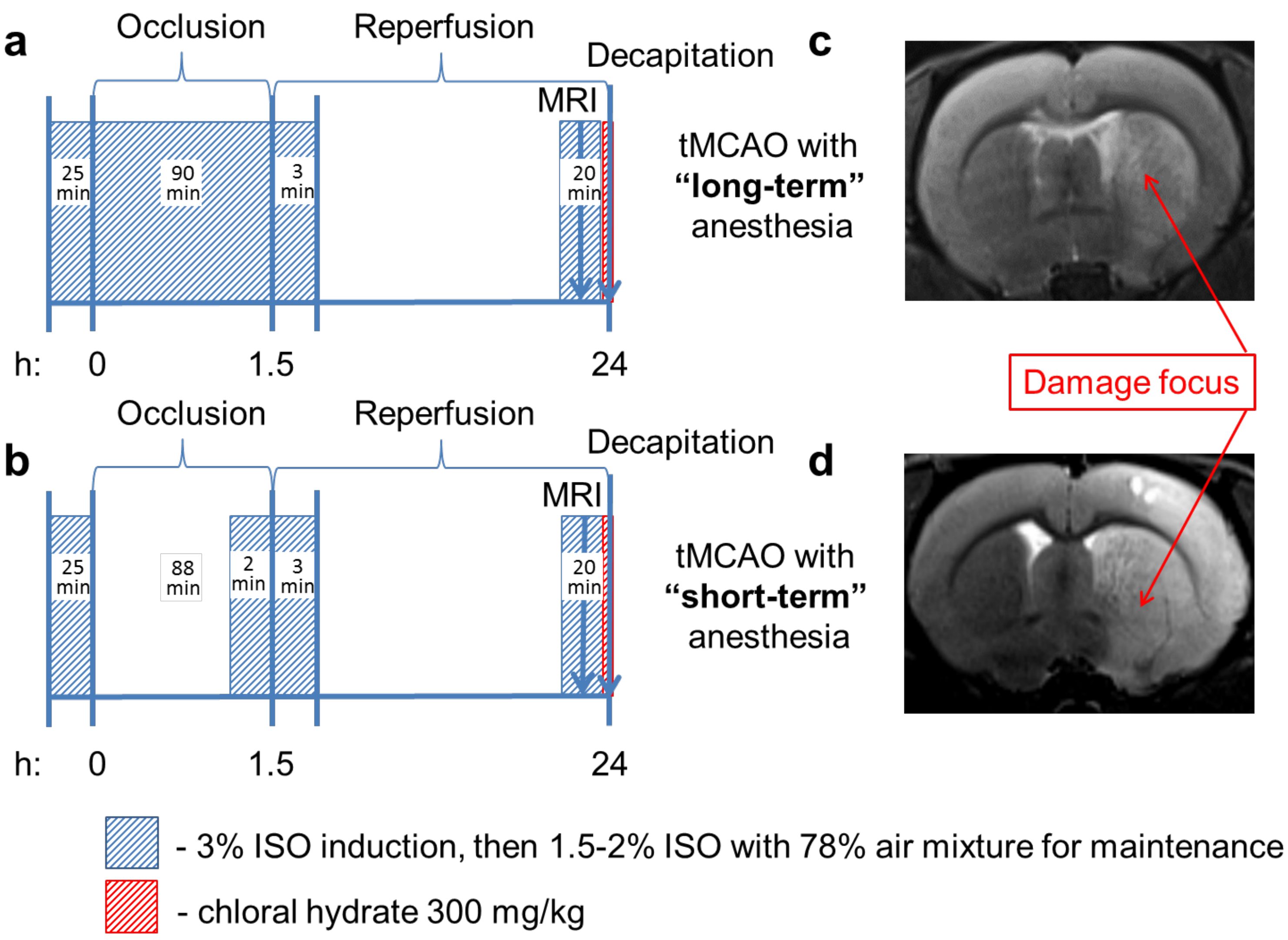

(**a**,**b**) tMCAO under "long-term" (**a**) and "short-term" (**b**) anesthesia are shown. In the rat tMCAO model, the time intervals between the onset of occlusion, reperfusion, MRI diagnostics, and decapitation of animals are indicated. Hatching marks the time spent by the animals under anesthesia with isoflurane and chloral hydrate. (**c**,**d**) MRI scans of the brain of ischemic rats after "long-term" (**c**) and "short-term" (**d**) anesthesia (about 10 min before decapitation) in the T2-WI mode are shown. A hyperintense MRI signal marks the area of ischemic injury.
